# Supplementary figures and images for: Long-term dietary nitrate supplementation does not reduce renal cyst growth in experimental autosomal dominant polycystic kidney disease
Source: PLoS One. 2021 Apr 22;16(4):e0248400. doi: 10.1371/journal.pone.0248400 (PMC8061912; doi:10.1371/journal.pone.0248400)

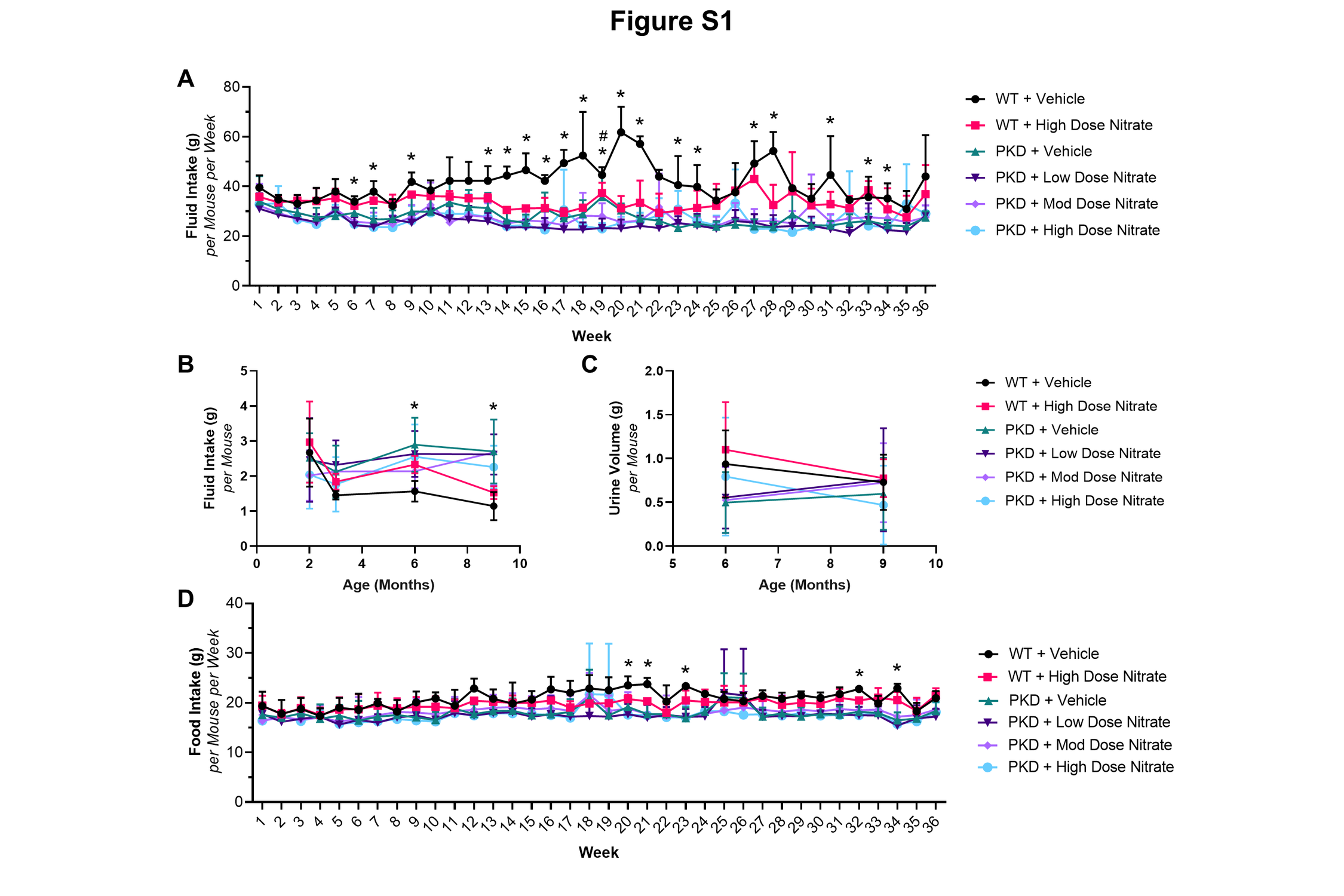

Supplement: S1 Fig — (A) Fluid intake by cage bottles measured weekly for 8 months; (B) Fluid intake by metabolic cage measured at 2, 3, 6 and 9 months of age; (C) Urine volume measured at 6 and 9 months of age; (D) Food intake measured weekly for 8 months. Data were combined from male and female mice (n = 4–6 per group per gender) and presented as means ± SD. *P<0.05 by one-way ANOVA. (TIF) [file pone.0248400.s001.tif]

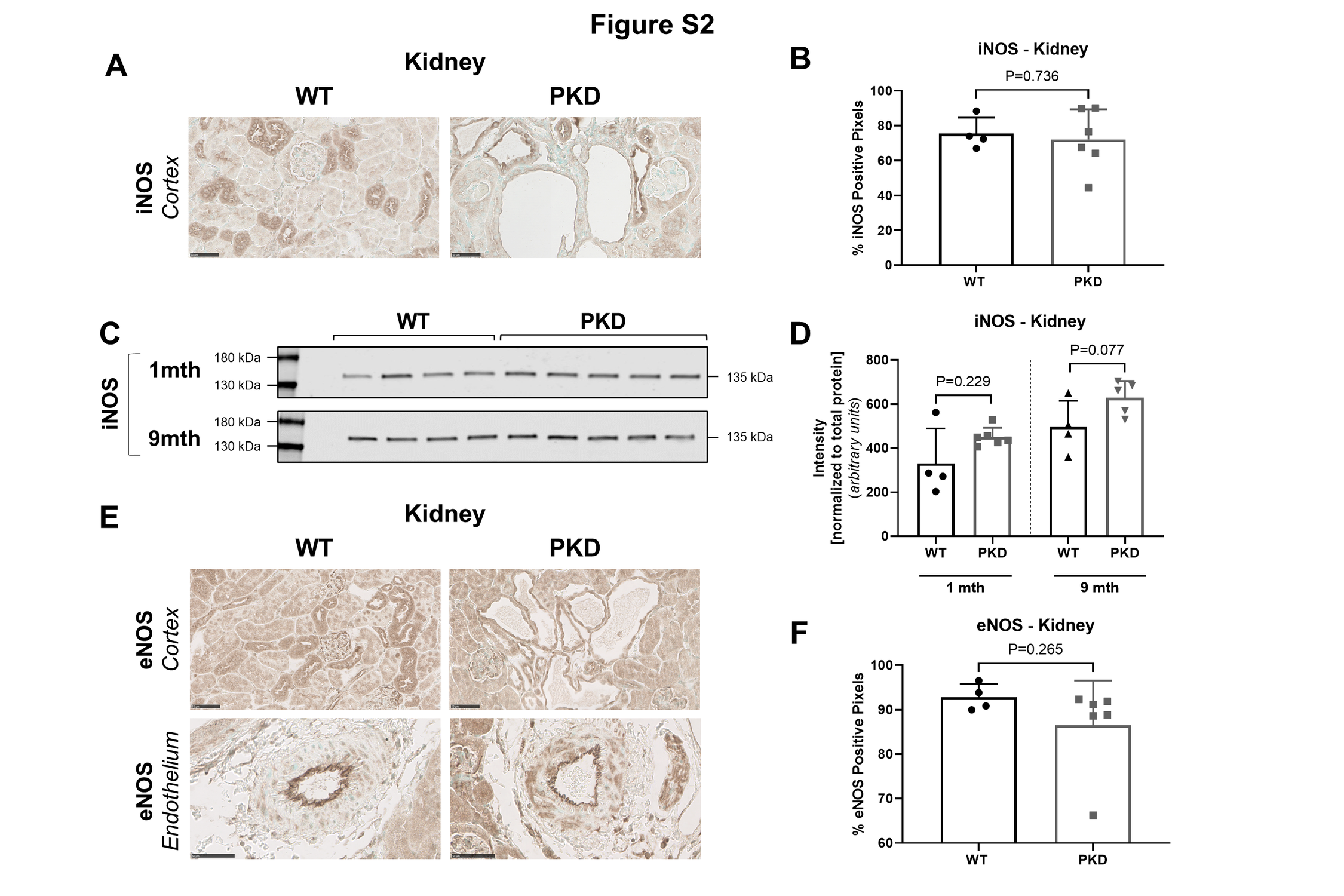

Supplement: S2 Fig — (A) Representative images of iNOS positive staining in mouse kidneys; (B) Quantification of iNOS positive staining in mouse kidneys; (C) Representative western blots of iNOS in mouse kidneys; (D) Quantification of renal iNOS in mouse kidneys by western blot; (E) Representative images of eNOS positive staining in mouse kidneys; (F) Quantification of eNOS positive staining in mouse kidneys. Data presented as means ± SD (n = 4–6 per group). (TIF) [file pone.0248400.s002.tif]

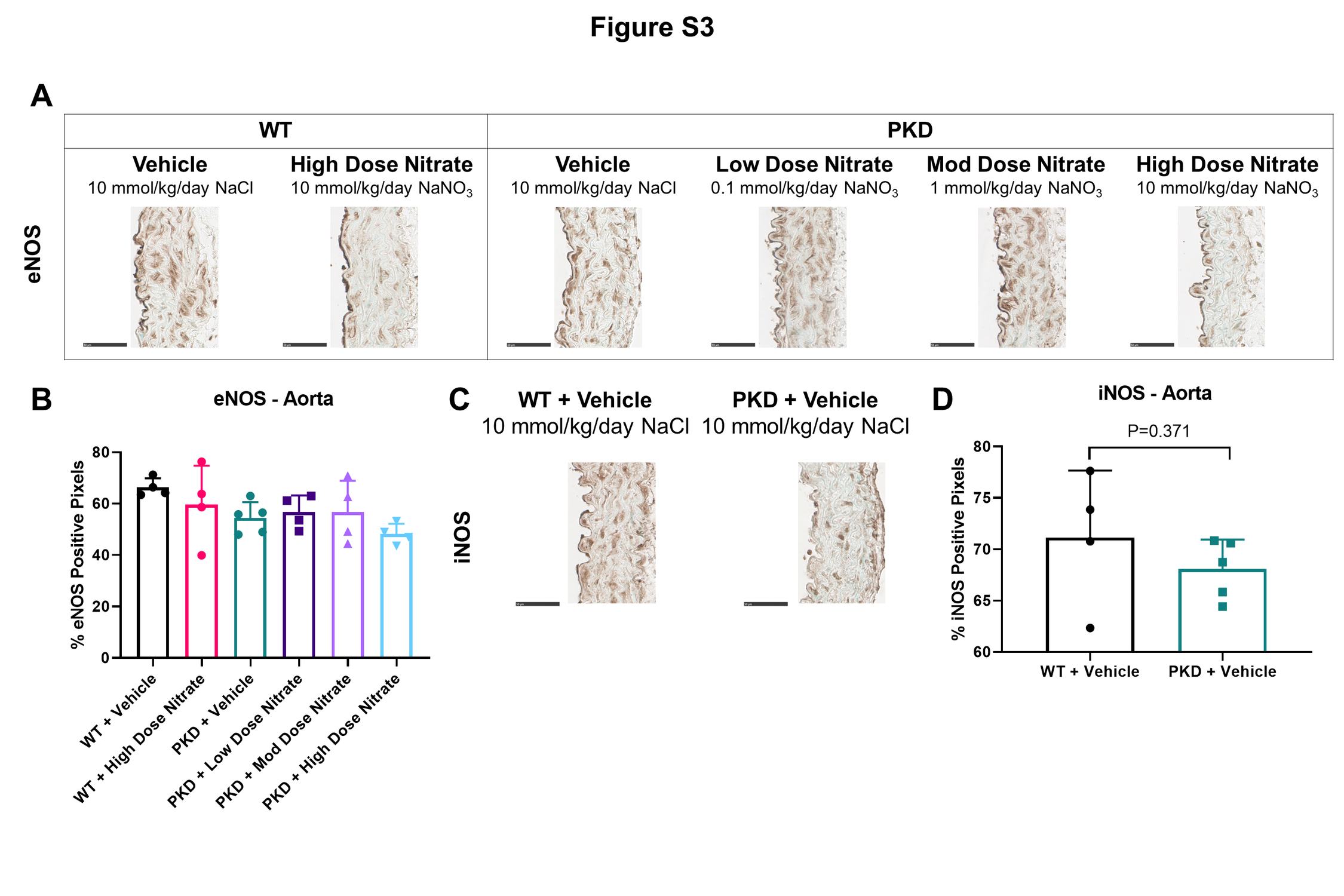

Supplement: S3 Fig — (A) Representative images of eNOS positive staining in mouse aorta; (B) Quantification of eNOS positive staining in mouse aorta; (C) Representative images of iNOS positive staining in mouse aorta; (D) Quantification of iNOS positive staining in mouse aorta. Data presented as means ± SD (n = 4–6 per group). (TIF) [file pone.0248400.s003.tif]

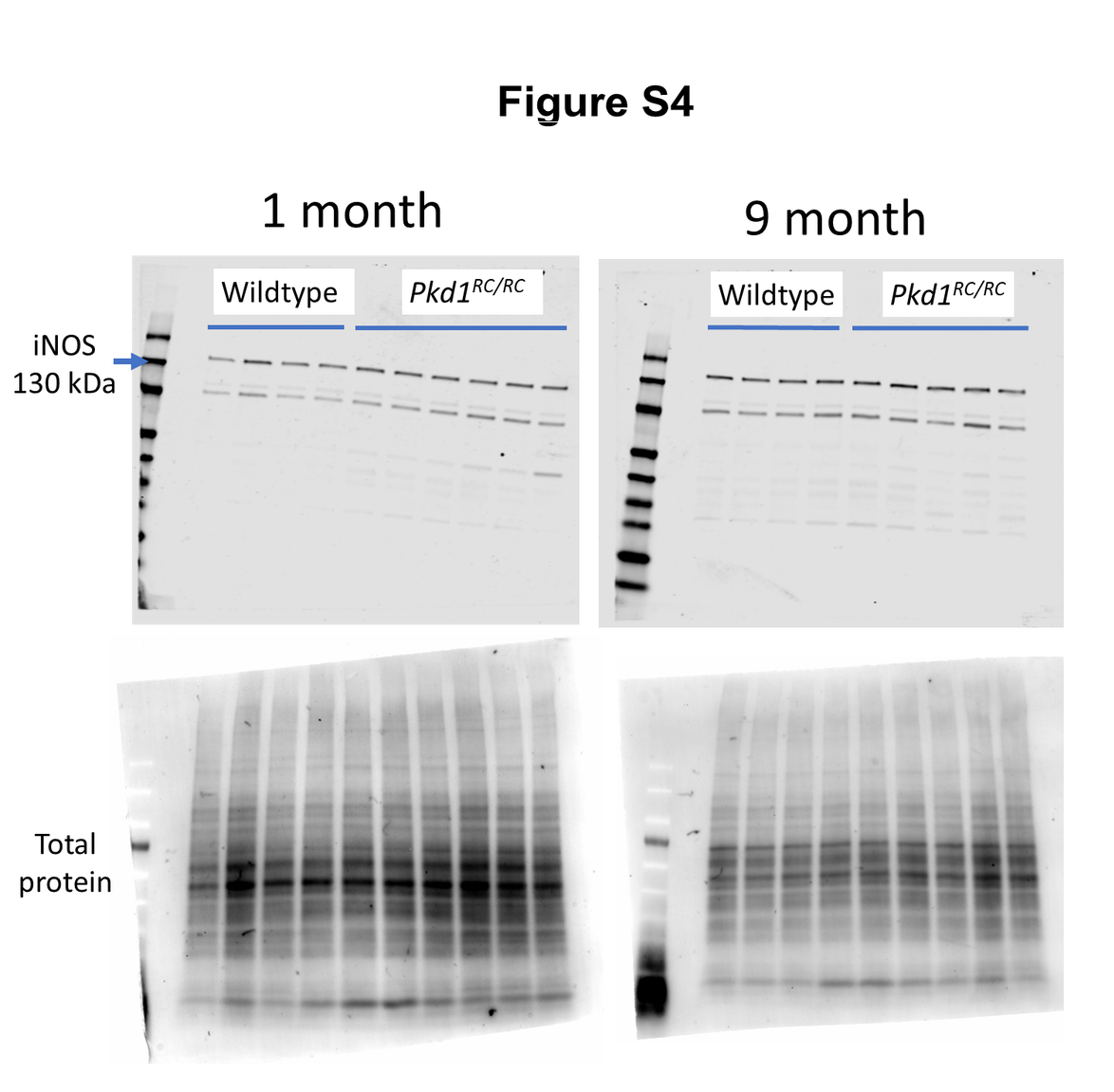

Supplement: S4 Fig — Top panels show immunological detection of iNOS and bottom panels show total protein as obtained by Stain-Free imaging technology (Bio-Rad Laboratories). (TIF) [file pone.0248400.s004.tif]

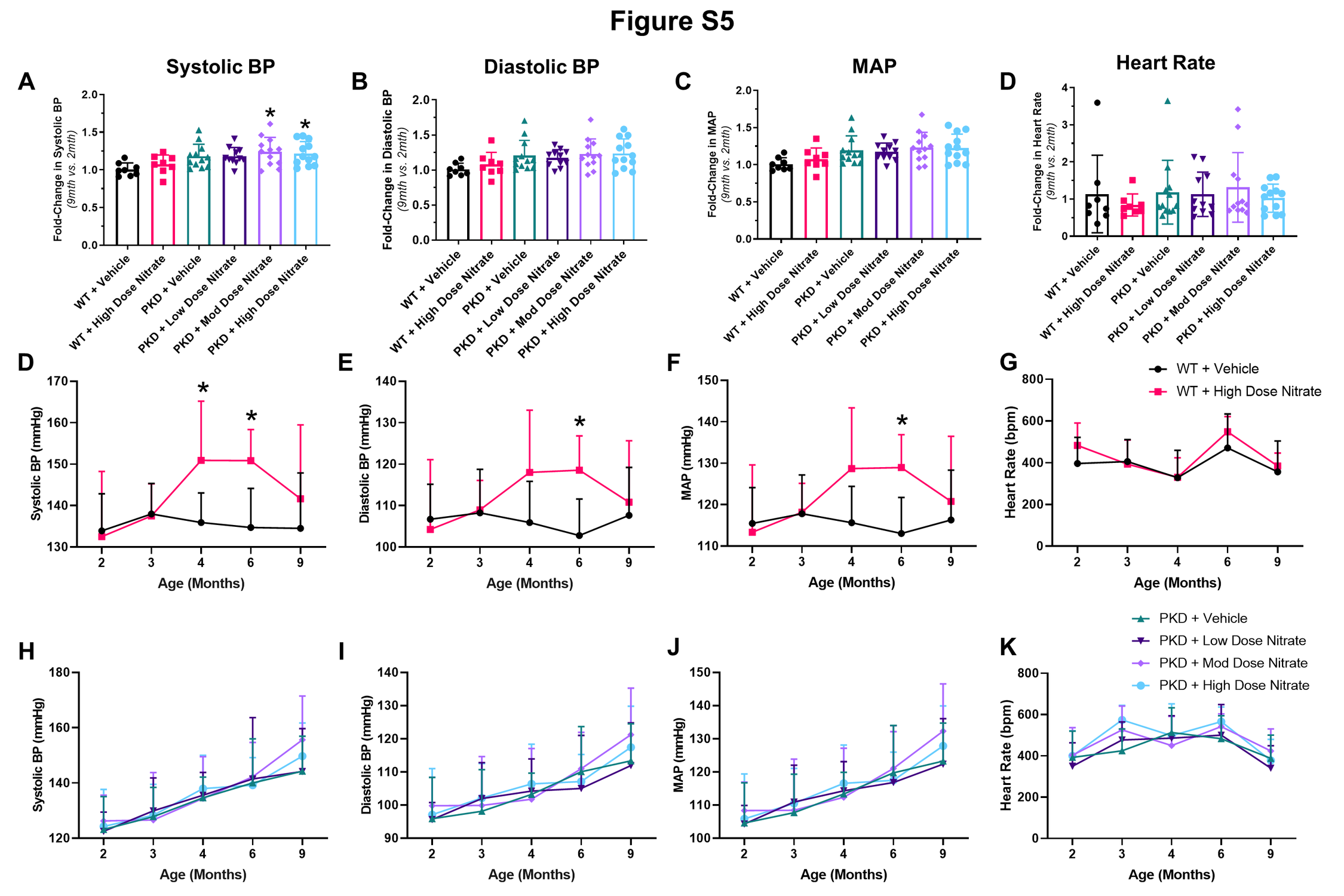

Supplement: S5 Fig — Panels (A), (B), (C) and (D) show fold-change in systolic (D & H), diastolic (E & I), mean arterial blood pressure (F & J) and heart rate (G & K) at 9 months of age compared to 2 months. *P<0.05 compared to WT + Vehicle by one-way ANOVA, followed by post-hoc analysis with the Tukey Kramer HSD test. (D & H) Time-course of systolic blood pressure; (E & I) Time-course of diastolic blood pressure; (F & J) Time-course of mean arterial pressure; and (G & K) Time-course of heart rate. *P<0.05 by one-way ANOVA. Data were combined from male and female mice (n = 4–6 per group per gender) and presented as means ± SD. (TIF) [file pone.0248400.s005.tif]

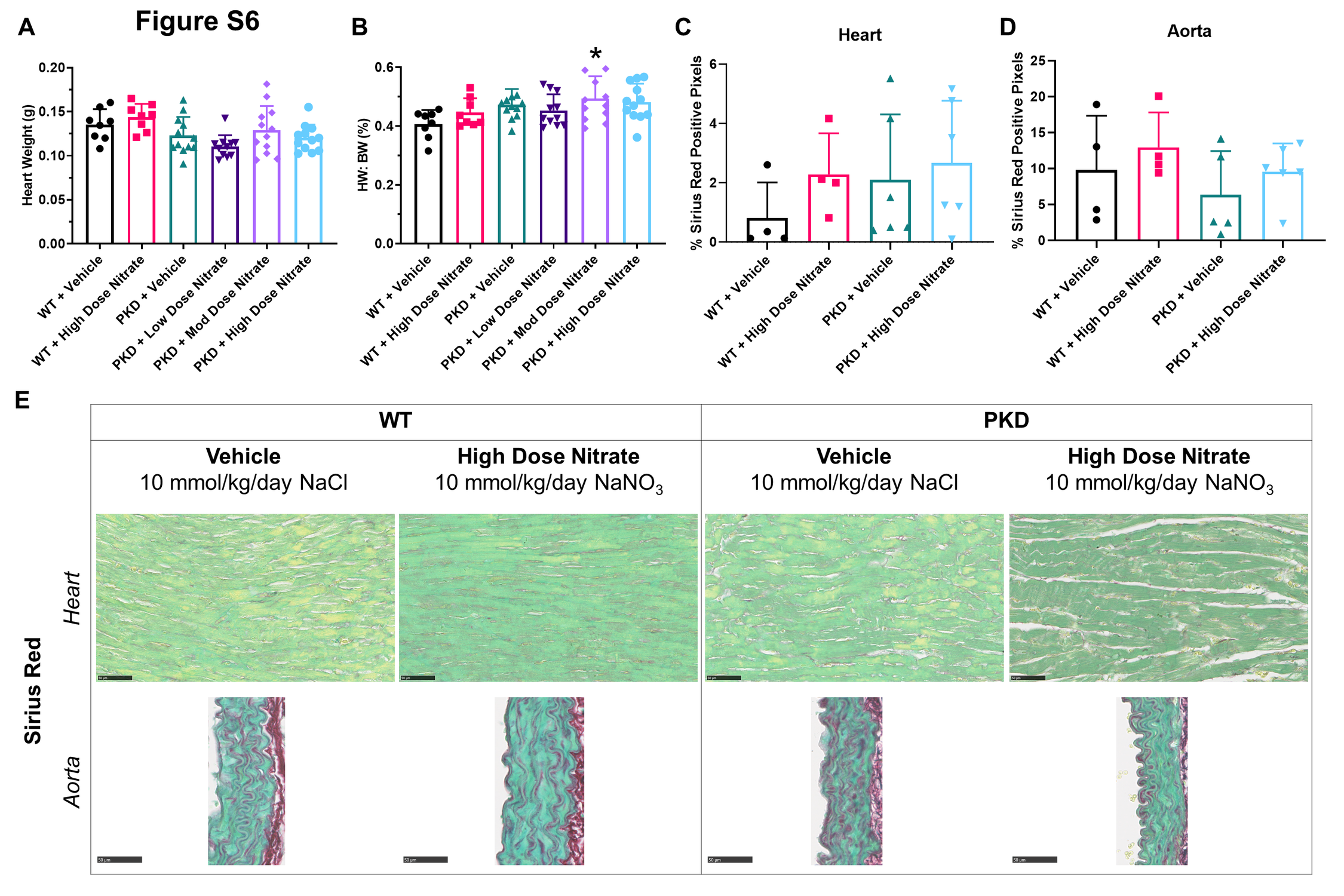

Supplement: S6 Fig — (A) Heart weight; (B) Heart weight to body weight ratio (HW: BW). Data were combined from male and female mice (n = 4–6 per group per gender) and presented as means ± SD. (C) Quantification of Sirius Red positive staining in mouse heart. (D) Quantification of Sirius Red positive staining in mouse aorta. Data presented as means ± SD (n = 4–6 per group). (E) Representative images of Sirius Red deposition in heart and aorta. (TIF) [file pone.0248400.s006.tif]
