# Supplementary material for: Long-term dietary nitrate supplementation does not reduce renal cyst growth in experimental autosomal dominant polycystic kidney disease
Source: PLoS One. 2021 Apr 22;16(4):e0248400. doi: 10.1371/journal.pone.0248400 (PMC8061912; doi:10.1371/journal.pone.0248400)
Supplement: S1 Table — (DOCX) [file pone.0248400.s007.docx]

**S1 Table. Body weight, kidney enlargement and percentage cyst area in wild-type (WT) and *Pkd1^RC/RC^* (PKD) mice treated with sodium chloride (vehicle) or sodium nitrate (low, 0.1 mmol/kg/day; moderate, 1 mmol/kg/day; high dose, 10 mmol/kg/day) for 8 months, sub-analyzed by gender.**

| **Group** | **Gender** | **n** | **Body Weight (g)** | **Two Kidney Weight (g)** | **KW: BW (%)** | **Cyst Area (%)** |
| --- | --- | --- | --- | --- | --- | --- |
| *WT + Vehicle* | M | 4 | 35.2 ± 3.2 | 0.34 ± 0.03 | 0.96 ± 0.06 | 4.2 ± 2.3 |
|  | F | 4 | 31.4 ± 1.4 | 0.25 ± 0.00 | 0.81 ± 0.03 | 7.1 ± 1.8 |
| *WT + High Dose Nitrate* | M | 4 | 33.3 ± 1.6 | 0.33 ± 0.02 | 1.00 ± 0.06 | 5.6 ± 2.4 |
|  | F | 4 | 31.3 ± 2.6 | 0.25 ± 0.02 | 0.81 ± 0.06 | 6.4 ± 1.0 |
| *PKD + Vehicle* | M | 6 | 29.3 ± 1.9 | 0.49 ± 0.05 | 1.68 ± 0.10 | 17.1 ± 4.9 |
|  | F | 6 | 22.8 ± 0.9* | 0.41 ± 0.08 | 1.79 ± 0.28 | 19.4 ± 6.4 |
| *PKD + Low Dose Nitrate* | M | 5 | 27.8 ± 1.2 | 0.40 ± 0.03 | 1.43 ± 0.11 | 14.1 ± 4.0 |
|  | F | 6 | 21.9 ± 1.0* | 0.37 ± 0.05 | 1.69 ± 0.22 | 20.6 ± 1.9 |
| *PKD + Mod Dose Nitrate* | M | 6 | 29.3 ± 1.6 | 0.51 ± 0.05 | 1.72 ± 0.10 | 15.7 ± 3.8 |
|  | F | 6 | 22.9 ± 1.1* | 0.43 ± 0.08 | 1.88 ± 0.38 | 22.5 ± 4.5 |
| *PKD + High Dose Nitrate* | M | 6 | 29.1 ± 1.8 | 0.52 ± 0.06 | 1.80 ± 0.21 | 20.4 ± 3.6 |
|  | F | 6 | 21.5 ± 0.8* | 0.38 ± 0.06* | 1.74 ± 0.20 | 14.7 ± 7.5 |

Data presented as means ± SD. *P<0.05 compared to type and group matched males by one-way ANOVA, followed by post-hoc analysis with the Tukey Kramer HSD test.
