# Supplementary material for: Long-term dietary nitrate supplementation does not reduce renal cyst growth in experimental autosomal dominant polycystic kidney disease
Source: PLoS One. 2021 Apr 22;16(4):e0248400. doi: 10.1371/journal.pone.0248400 (PMC8061912; doi:10.1371/journal.pone.0248400)
Supplement: S2 Table — (DOCX) [file pone.0248400.s008.docx]

**S2 Table. Serum urea in wild-type (WT) and *Pkd1^RC/RC^* (PKD) mice treated with** **sodium chloride (vehicle) or sodium nitrate (low, 0.1 mmol/kg/day; moderate, 1 mmol/kg/day; high dose, 10 mmol/kg/day) for 8 months.**

| **Group** | **Serum urea (mmol/L)** |
| --- | --- |
| *WT + Vehicle* | 11.1 ± 1.6 |
| *WT + High Dose Nitrate* | 10.5 ± 1.2 |
| *PKD + Vehicle* | 10.7 ± 1.1 |
| *PKD + Low Dose Nitrate* | 11.8 ± 2.8 |
| *PKD + Mod Dose Nitrate* | 11.2 ± 2.3 |
| *PKD + High Dose Nitrate* | 11.0 ± 0.6 |

Data presented as means ± SD (n=4-6 per group).
