# Supplementary material for: Long-term dietary nitrate supplementation does not reduce renal cyst growth in experimental autosomal dominant polycystic kidney disease
Source: PLoS One. 2021 Apr 22;16(4):e0248400. doi: 10.1371/journal.pone.0248400 (PMC8061912; doi:10.1371/journal.pone.0248400)
Supplement: Attachment — Submitted filename: Review Comments.docx [file pone.0248400.s009.docx]

**Overall summary:**

This manuscript tested the hypothesis that NO deficiency is feature of ADPKD, and that long-term oral nitrate supplementation reduces cardiovascular and cystic kidney disease progression in a genetic ortholog of ADPKD (Pkd1 RC/RC mice). In their analyses, a human ADPKD tubular cell line, NO was reduced by 75% to 73%, compared to controls. In Pkd1 RC/RC mice, the expression of aortic endothelial NO synthase (NOS) was decreased by 18%. Moreover, in the three-dimensional MDCK in vitro cyst model, treatment with NO substrates (L-arginine and sodium nitroprusside) reduced cyst growth by up to 18%. In Pkd1 RC/RC mice, sodium nitrate supplementation increased serum nitrate/nitrite levels by ~25-fold compared to control, with no adverse effects. However, sodium nitrate supplementation for 8 months, regardless of dosage, did not alter the progression of cardiovascular (assessed by tail cuff systolic blood pressure, ex vivo aortic wire myography and cardiac fibrosis) or cystic kidney disease in Pkd1 RC/RC mice. In conclusion, their data suggested that NO synthesis is reduced in ADPKD, but the long-term intake of oral nitrate in drinking water is not sufficient to correct this deficiency and attenuate cardiovascular and kidney disease progression. Overall, while this article is interesting and could potentially be of significance in ADPKD field, there are significant points that the authors could clarify better. At this stage, this article would benefit from a fair amount of editing before publishing.

**Major comment:**

1. To test their hypothesis of oral nitrate, to reduce cardiovascular and kidney diseases progression, authors didn’t establish reliable control before executing their animal model experiments. Despite the fact that oral nitrate has been shown to reduce blood pressure (J Appl Physiol (1985). 2019 Oct 1;127(4):1085-1094.), Wild-type (control) treated with high dose of nitrate had an increase blood pressure (Figure 6 D-F). This raises the question of the purity and/or the nitrate supplement that was used in this study. Or, perhaps the (PKD1 RC/RC mouse model) have an impaired microbiota in bioactivation of dietary nitrate (Free Radic Biol Med. 2019 Dec;145:342-348.).

**Minor comments:**

1. Page 14: Figure 1 need to be mentioned in the result section before start interpreting Figure 2.
2. Page 15, Line 282: Please delete the duplication: “P<0.05 compared to compared to…”
3. Figure 3 and Figure 4: kidney tissue Images are very poor quality that is not easy to asses.
4. Figure 4 E-F: It is strange that the authors have examined the expression of iNOS (4 c-d) and kind of ignored all together the expression of eNOS to validate their immunohistochemistry results.
5. Flow of the figures and results is hard to follow, and need be to more organize. For example, Supplemental figures are not in order throughout the manuscript (S1 vs S2 and so on).
6. Table 1: There is no rational, or nothing was given by authors, is to why they selected MCDK cell line over WT9-7 and/or WT9-12, nor any justification of selected concentrations.
7. Page 17, Line 329: Again please delete the duplication “P<0.05 compared to compared to…”
8. Figure 5: Again, inconsistency of representing the results. Kidney weight and kidney to body weight ratio are given, but not the same with heart. Also, authors in (Fig 5C-E) are over interpreting these data, Page 17, Line 336, “Continuous treatment with NaNO3 for 8 months…did not alter the progression of kidney or heart enlargement in Pkd1RC/RC mice.” For example, in figure 5C low dose of Nitrate seems to have directly reduced the kidney enlargement, despite the KW: BW. In the contrary, moderate dose of Nitrate had at least increased heart to body weight ratio. This further might suggest that Nitrate effect might be dose dependent. Have the authors at least sub-analyzed these data by gender?
9. Page 17, Line 363: confusing statement and perhaps incorrect. “Neither tail-cuff blood pressure nor heart rate were altered by long-term NaNO3 supplementation in wild-type or Pkd1RC/RC mice (Fig 6A-I & S3A-C Fig).” First, Blood pressure of Wild-type had indeed been negatively altered by long-term NaNO3 (Figure 6 D-F), particularly in the 6^th^ month. Second, again authors need to explain that how high dose of nitrate is increasing BP rather than decreasing it in their control. This raises the questions of the purity and/or the supplement that was administered to the mice. Furth, Please move more relevant heart rate supplemental result to the main figure.
