# Supplementary material for: Long-term dietary nitrate supplementation does not reduce renal cyst growth in experimental autosomal dominant polycystic kidney disease
Source: PLoS One. 2021 Apr 22;16(4):e0248400. doi: 10.1371/journal.pone.0248400 (PMC8061912; doi:10.1371/journal.pone.0248400)
Supplement: Attachment — Submitted filename: 2. Response to Reviewers.docx [file pone.0248400.s011.docx]

**Response to Editor’s Comments**

**Editor Comment #1:** *Reviewer #1 still has several issues that you need to address. They all relate to the validity of your conclusions with respect to ADPKD considering 1) the mild phenotype of your model and 2) your extrapolation from findings in a cultured cell model. Your conclusions relating to in vivo effects of nitrates in ADPKD need to be (further) toned down.*

Response: Thank you for the opportunity to further improve this manuscript. We agree and have extensively revised the entire manuscript. For the *in vivo* study in the *Pkd1^RC/RC^* mice, we have now placed all emphasis on the efficacy of nitrates on renal cyst growth. Furthermore, due to the lack of cardiovascular disease phenotype in the *Pkd1^RC/RC^* mouse, we agree, and have stated throughout the manuscript, that it is not possible to conclude whether nitrates have efficacy on this complication (see Abstract: p3, Lines 43-46; Results: p18, Lines 377-380; and Discussion: p23-24, Lines 495-497).

**Editor Comment #2:** *Reviewer #1 also still finds the manuscript somewhat chaotic and I agree. The manuscript will have to be (partly) restructured. Please present ALL in vitro data first and then present your study characterising NOS expression in untreated ADPKD and wild-type mice. Next, use ALL these data to motivate your intervention study. Most of the figures relating to your intervention study show no effects of the intervention and can be moved to the supplement.*

Response: We agree, and the entire manuscript has now been extensively re-written and edited, and the Results have also been partly re-structured. As suggested, all *in vitro* data is presented first followed by *in vivo* data. Figures related to cardiovascular phenotype have moved to the Supporting Information (see Figs S6-8).

**Editor Comment #3:** *More emphasis in the discussion should be on the in vitro findings and less on the intervention study.*

Response: We agree, and the Discussion has also been extensively edited. More detailed discussion is provided on the *in vitro* findings (see Discussion: p21-22, Lines 428-460), and we have reduced the sections on the intervention study (see Discussion: p22-23, Lines 470-485).

**Response to Reviewer #1 Comments**

**Reviewer Comment #1:** *My main concern is that there are no changes in the cardiovascular phenotype between PKD and control. Authors postulate that this is a very mild phenotype. The way the data is presented, there are no cardiovascular changes between wild type and PKD. On some level, the conclusion that there are no effects on cardiovascular phenotype is true, however I do not think it should be concluded that nitrates do not affect the cardiovascular outcomes (we do not see any on the and treated group) in PKD.*

Response: We thank Reviewer #1 for emphasising this point and we agree. Consequently, we have extensively revised the entire manuscript. Specifically, for the *in vivo* data in the *Pkd1^RC/RC^* mouse model, we placed all emphasis on the efficacy of nitrates on renal cyst growth. Furthermore, , due to the lack of cardiovascular disease phenotype in the *Pkd1^RC/RC^* mouse model, we have explicitly stated that it is not possible to conclude whether nitrates have efficacy on this complication (see Abstract: p3, Lines 43-46; Results: p18, Lines 377-380; and Discussion: p23-24, Lines 495-497).

**Reviewer Comment #2:** *To my read, the change in nitric oxide is supported by the DAF data (cell culture of human cells) and is not related to the ADPKD model. If that is the case, the conclusion that there is a decrease in nitric oxide in the RCRC murine model of PKD is not supported. Please clarify if I am missing something.*

Response: We thank Reviewer #1 for this comment. The manuscript has been revised to emphasise the relevance of the human ADPKD cell line data as well as the *in vitro* cyst model (see Introduction: p5, Lines 88-91; Materials and Methods: p7, Lines 125-127; Discussion p21-22, Lines 428-460). Furthermore, urinary nitrate excretion in PKD1 mice was reduced compared to wild-type mice (see Results: p16, Lines 326-327; Table 2), suggesting that nitric oxide is attenuated in this model, and we have highlighted this data, as it was not discussed in the previous version.

**Reviewer Comment #3:** *I feel that too much data is presented together, cystic renal changes together with cardiovascular changes that are not truly there and the paper altogether appears to diffuse. Consider focusing on the cystic changes.*

Response: We agree, and the entire manuscript has now been extensively re-written and edited, and the Results have also been partly re-structured. For the *in vivo* study in the *Pkd1^RC/RC^* mice, we have now placed all emphasis on the efficacy of nitrates on renal cyst growth. The Results text related to cardiovascular disease outcomes has been significantly condensed and figures have moved to the Supporting Information (see Results: p18, Lines 377-380; Figs S6-8).

**Reviewer Comment #4:** *The data on the renal tubular cell line is not from the specific PKD model mentioned in the title and is somewhat out of place.*

Response: We have enhanced our explanation of the rationale for studies conducted in the ADPKD cell lines (see Introduction: p5, Lines 88-91), and also revised the title to refer to “experimental ADPKD” so that the clarity of studies performed is improved.

**Reviewer Comment #5:** *Figure 1. What is the effect of nitrates on the cell line?*

Response: This is an interesting point and was also raised by the research team during the planning of the study. We did not investigate this hypothesis because conversion of nitrate to NO *via* the entero-salivary pathway is dependent on the presence of oral and gut bacteria [1-4] (see Discussion: p23, 488-492). Therefore, it would not be possible to easily replicate this process in our simple single-cell based cell culture model. However, it would interesting to consider this in future studies, should oral nitrates be shown to have efficacy on cardiovascular disease in other PKD models. In any case, our *in vivo* studies demonstrated that there was no efficacy on kidney cyst growth, and we feel that this is the most important finding.

**References**

1.	Tiso M, Schechter AN. Nitrate reduction to nitrite, nitric oxide and ammonia by gut bacteria under physiological conditions. PLOS One. 2015;10(3):e0119712-e. doi: 10.1371/journal.pone.0119712. PubMed PMID: 25803049.

2.	Bondonno CP, Liu AH, Croft KD, Considine MJ, Puddey IB, Woodman RJ, et al. Antibacterial Mouthwash Blunts Oral Nitrate Reduction and Increases Blood Pressure in Treated Hypertensive Men and Women. Am J Hypertens. 2015;28(5):572-5. doi: 10.1093/ajh/hpu192.

3.	Goh CE, Trinh P, Colombo PC, Genkinger JM, Mathema B, Uhlemann AC, et al. Association Between Nitrate-Reducing Oral Bacteria and Cardiometabolic Outcomes: Results From ORIGINS. J Am Heart Assoc. 2019;8(23):e013324. Epub 2019/11/27. doi: 10.1161/jaha.119.013324. PubMed PMID: 31766976; PubMed Central PMCID: PMCPMC6912959.

4.	Moretti C, Zhuge Z, Zhang G, Haworth SM, Paulo LL, Guimarães DD, et al. The obligatory role of host microbiota in bioactivation of dietary nitrate. Free Radic Biol Med. 2019;145:342-8. doi: 10.1016/j.freeradbiomed.2019.10.003.
