# Supplementary material for: Long-term dietary nitrate supplementation does not reduce renal cyst growth in experimental autosomal dominant polycystic kidney disease
Source: PLoS One. 2021 Apr 22;16(4):e0248400. doi: 10.1371/journal.pone.0248400 (PMC8061912; doi:10.1371/journal.pone.0248400)
Supplement: Attachment — Submitted filename: 2. Response to Reviewers comments.docx [file pone.0248400.s012.docx]

**Response to Editor’s Comments**

**Editor Comment #1.** *More changes are required, particularly in the abstract. You really must take the cardiovascular aspect out of the paper to merit acceptance. Make sure that you adequately address all the remaining comments.*

***Author Response:*** We have removed the cardiovascular aspect of the data (aortic myography) from all the sections (See Abstract p3. Lines 43-46, Introduction p4. Lines 62-67, Methods p9. Lines 179-180; pp12-13, Lines 243-277, Results p18, Lines 377-380, Discussion pp.23-24 Lines 495-497; Supplemental Figures S1 and S7).

**Response to Reviewer’s Comments**

**Reviewer Comment #1.** *The majority of the introduction has to do with endothelial dysfunction and vascular changes but again is not the focus of the paper in this version (neither on the title nor the results section). In other words I do not see how the introduction leads to the test of nitrates on cyst growth.*

***Author Response:*** We have removed the section of the Introduction from p4 (Lines 62-67) where the endothelial dysfunction in ADPKD was discussed.

**Reviewer Comment #2.** *I appreciate the toning down of the cardiovascular data in this version. However, I do not think that they have a role in this manuscript. I would recommend removal of the sentence in the abstract starting with “the phenotype of cardiovascular disease…” as well as the myography and other CV data is not relevant here. BP may provide support for the mild nature of this model, however I would remove the use of nitrates on cardiovascular outcomes (there are none on the disease model).*
***Author Response:*** We have removed the cardiovascular data from all the sections of the manuscript (See Abstract p3. Lines 43-46, Introduction p4. Lines 62-67, Methods p9. Lines 179-180; pp12-13. Lines 243-277, Results p18. Lines 377-380, Discussion pp.23-24. Lines 495-497; Supplemental Figures S1, S6-S8).

**Reviewer Comment #3.** *Page 18. See comment 2.*

***Author Response:*** The results on cardiovascular phenotype (p18. Lines 377-380) have been removed.

**Reviewer Comment #4.** *Figure 6. I do not see any quantification differences. If there are some on the staining, they should be made more clear the graphics.*

***Author Response:*** There were no quantitative differences in either nitrotyrosine or γH2AX expression in the wildtype or Pkd1RC/RC mice treated with NaNO3 compared to vehicle (Panels B to D).

**Reviewer Comment #5.** *Could be that the decrease in urinary nitrate reflect more than just a decrease in NO bioavailability? Renal function? It is good to see that the animal that receive nitrates have higher levels in the urine, demonstrating that the actually received the nitrates. I am not sure that this piece of data reinforces the hypothesis. Please clarify if I am missing something.*

***Author Response:*** Thank you for raising this important question. The renal functions was a part of our initial manuscript (but removed as per reviewer suggestion following first revision). Briefly, this data showed that serum urea in *Pkd1RC/RC* mice was not different to wildtype mice. On contrary, urinary nitrate in the *Pkd1RC/RC* mice increased with increasing nitrate supplementation strengthening the fact that renal filtration of nitrates is normal in the *Pkd1RC/RC* mice, and the lower nitrate in urine of *Pkd1RC/RC* mice (~30% of wildtype) supports the hypothesis that NO metabolism is reduced in the Pkd1RC/RC model.
