# Supplementary material for: Long-term dietary nitrate supplementation does not reduce renal cyst growth in experimental autosomal dominant polycystic kidney disease
Source: PLoS One. 2021 Apr 22;16(4):e0248400. doi: 10.1371/journal.pone.0248400 (PMC8061912; doi:10.1371/journal.pone.0248400)
Supplement: Attachment — Submitted filename: 2. Response to Reviewers Comments.docx [file pone.0248400.s013.docx]

**Response to Comments of the Editors and Reviewers**

**Editorial Comments:**

Please address ALL remaining comments of reviewer #1 and also delete text on cardiovascular disease:

- Keywords (line 48)
- Aims (line 87)
- Discussion, last sentence (lines 460-461)

*Authors Response*: Thank you. All text referring to cardiovascular disease has been removed, and all of the Reviewers comments have been addressed. Please see section below for further details.

**Response to Reviewer #1**

***Comment #1:*** *Figure 1: Please remove the aortic myography reference*

*Author Response*: Thank you. The reference to aortic myography has been removed from Figure 1.

**Comment #2:**  *Figure 6: authors observed that there is no change in their evaluation of the nitric oxide system in vivo between control in PKD. This is relevant because if we want to transfer the knowledge from cell culture to the in vivo setting, we would expect some sort of correlation between the endo findings which is not present. The fact that that correlation is not present, makes the cell cultures-in vivo translation a bit less relevant. That being said, this reviewer recognizes that authors did not do the same evaluation in cell culture and in vivo and thus a direct comparison is difficult to make. This issue must be adequately discussed.*

*Author Response:* Thank you for the comment. The experiments in Figure 6 were performed to assess whether oral nitrate consumption caused any of the adverse effects known to occur with excess intake. The latter includes oxidative stress (in particular nitrotyrosine formation) and DNA damage (gamma-H2AX). Our data showed that nitrate supplementation did not cause these mutagenic effects and was not an explanation for the lack of efficacy on kidney cyst growth. To address Reviewer #1 comments:

- the results section has been modified to improve clarity of this data (See lines 351-355); and
- that the differences between the design of the *in vivo* and *in vitro* models prevents direct comparison of the data between these experiments, has been discussed as a limitation of the current study (See lines 437-448).
